# Persistent Bacteremia Due to Escherichia coli Vertebral Osteomyelitis

**DOI:** 10.7759/cureus.52016

**Published:** 2024-01-10

**Authors:** Shinichi Kida, Yasushi Shibue

**Affiliations:** 1 Emergency Medicine, Yokohama City Minato Red Cross Hospital, Yokohama, JPN; 2 Infectious Diseases, Yokohama City Minato Red Cross Hospital, Yokohama, JPN

**Keywords:** blood stream infection, persistent bacteremia, escherichia coli, pyogenic osteomyelitis, pyogenic vertebral osteomyelitis

## Abstract

Vertebral osteomyelitis is a disc and vertebral infection that causes nonspecific symptoms such as back pain, fever, and weakness. The most common causative pathogen is *Staphylococcus aureus*, and *Escherichia coli *(*E. coli*) is an uncommon cause. An 88-year-old man presented to the emergency department with a fever and lower back pain. His blood cultures were persistently positive for *E. coli *on days one, three, and five, and a diagnosis of vertebral osteomyelitis was made after an MRI of the lumbar spine. It has been reported that infectious dissemination to the vertebrae may occur through Batson’s venous plexus, which is a network of paravertebral veins, and the pelvic venous plexus. Clinicians should remember that vertebral osteomyelitis can be a cause of persistent bacteremia.

## Introduction

Vertebral osteomyelitis is an infection of the disc and contiguous vertebrae that presents with back pain, fever, and weakness. These nonspecific symptoms pose a diagnostic challenge for clinicians, which may cause a significant delay in diagnosis (a range of 42-59 days has been reported) [1]. Its incidence is estimated at 2.4 cases per 100,000 people [2]; however, it is increasing due to aging populations, spinal surgery, and intravenous drug users [3]. The most frequent pathogens are *Staphylococcus aureus* and coagulase-negative staphylococci [4]. Although uncommon, *Escherichia coli* (*E. coli*) can cause vertebral osteomyelitis [5]. We present the case of a patient with persistent bacteremia due to *E. coli* vertebral osteomyelitis.

## Case presentation

**Figure 1:**
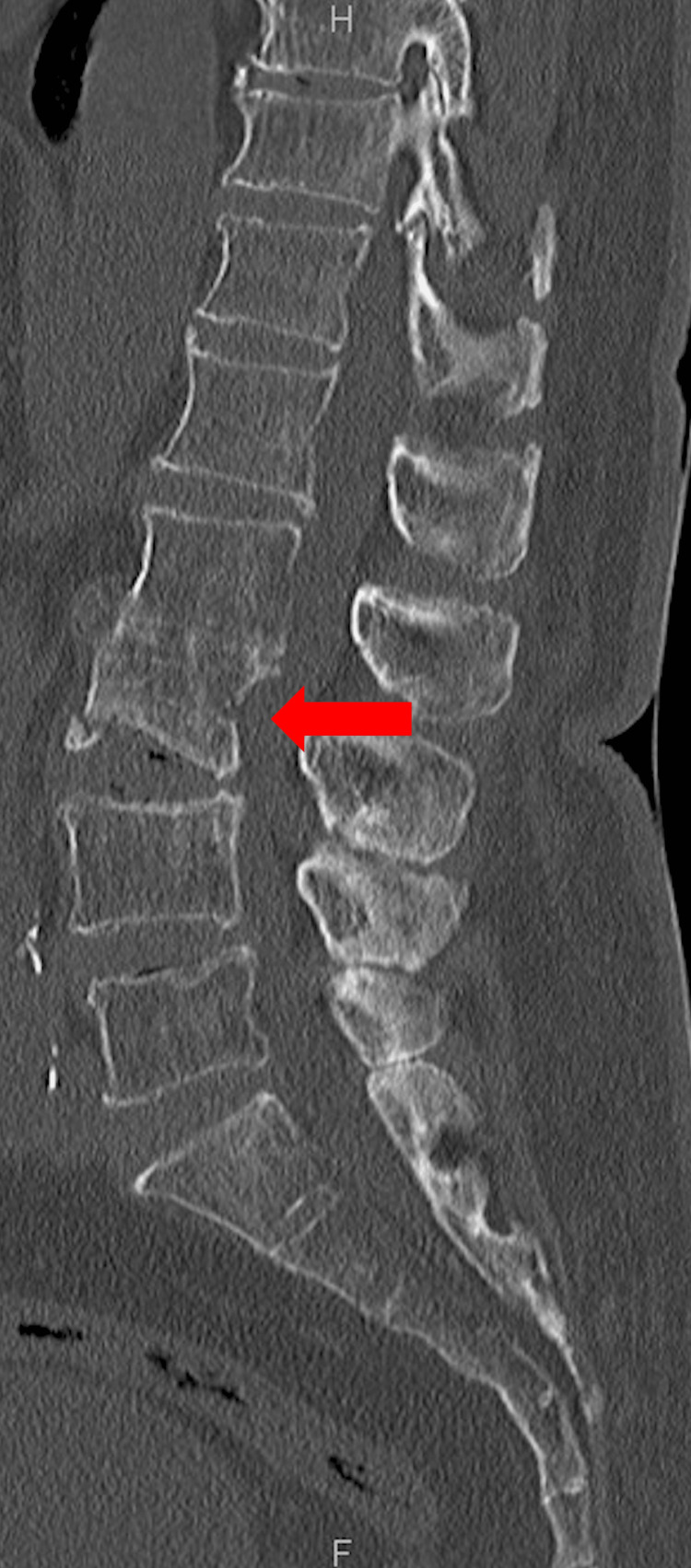
Computed tomography of the lumbar spine on arrival showed an L3 fracture (red arrow)

**Figure 2:**
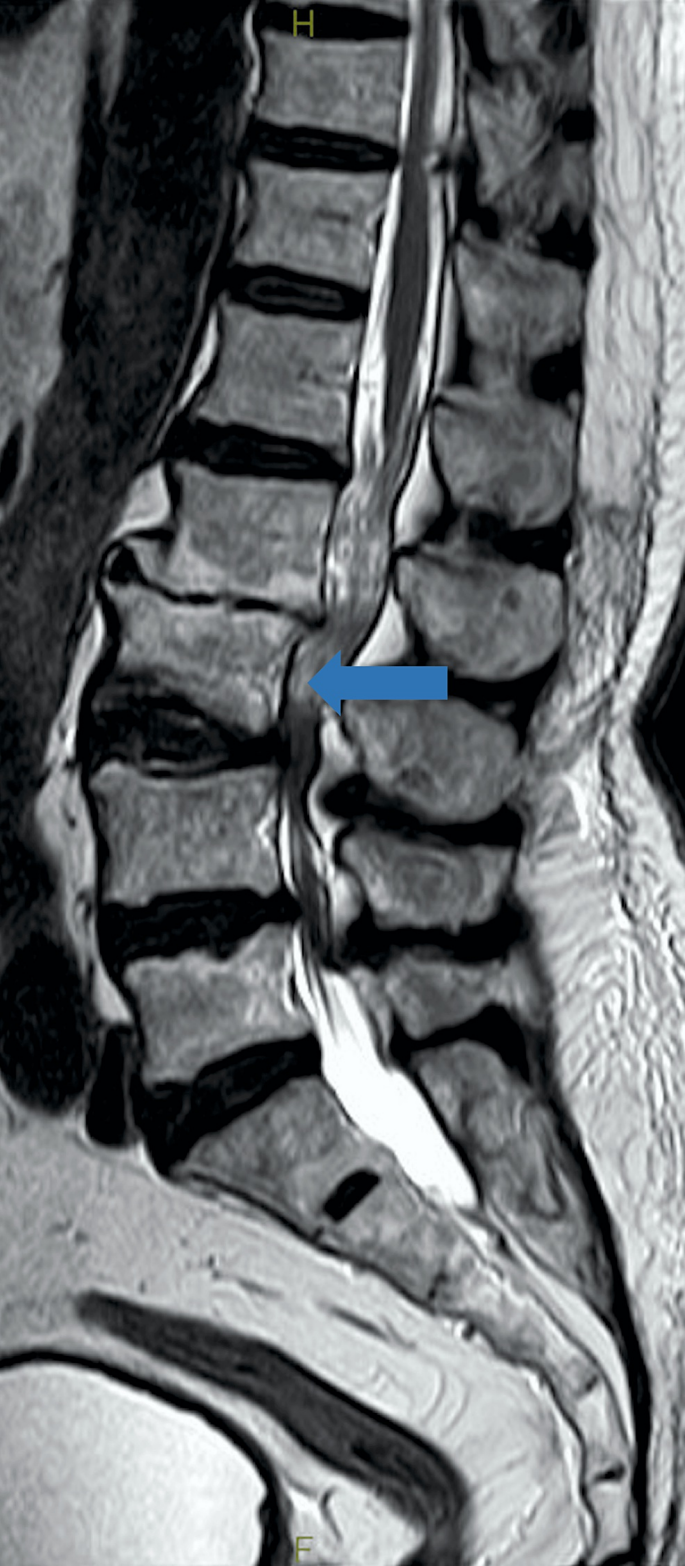
Magnetic resonance imaging of the lumbar spine on arrival revealed an L3 fracture (blue arrow).

An 88-year-old man with a history of dyslipidemia and hyperuricemia was transferred to the emergency department with a fever and low back pain. He had been healthy but reported that his lumbar pain started approximately four months prior after landing on his buttocks from a fall. His backache spontaneously worsened a month before his presentation. He fell again the day before visiting the hospital. On arrival, he exhibited rigors and was immobilized because of his backache. His regular medication included ursodeoxycholic acid, febuxostat, and ethyl icosapentate. He was not prescribed any antibiotics during this period. There was no history of dysuria, urgency, frequency, abdominal pain, diarrhea, or constipation. On examination, he was febrile, with a temperature of 40°C, a blood pressure of 118/56 mm Hg, a pulse rate of 85 beats/min, and a respiratory rate of 16 breaths/min with an oxygen saturation of 93% in room air. No conjunctival petechiae were observed. He had a systolic murmur at the apex, the lungs were clear on auscultation, and his abdominal examination was normal. The patient had no costovertebral or prostate tenderness and no neurological symptoms. Blood examination revealed mild C-reactive protein elevation (3.1 mg/L); however, a complete blood count, other chemistry tests, and urine analysis were normal. A CT scan and MRI of the lumbar spine were conducted because of concerns regarding vertebral infection. They showed an L3 compression fracture with no evidence of vertebral infection (Figures 1, 2).

He was admitted to the medical ward for the treatment of his back pain. However, the following day, his blood cultures proved positive for *E. coli*, while the urine culture was negative. Piperacillin-tazobactam was administered to treat infections of unknown origin. Although antibiotic susceptibility revealed pan-sensitive *E. coli *(Table 1), his blood cultures were still positive on days three and five.

**Table 1:** Blood cultures susceptibility profile and interpretation MIC: minimum inhibitory concentration; S: susceptible

	Interpretation	MIC (µg/mL)
Amikacin	S	<=16
Ampicillin	S	<=8
Ampicillin/Sulbactam	S	<=8
Aztreonam	S	<=4
Cefazolin	S	<=2
Cefepime	S	<=2
Cefmetazole	S	<=16
Ceftazidime	S	<=1
Ceftriaxone	S	<=1
Ciprofloxacin	S	<=1
Gentamicin	S	<=4
Levofloxacin	S	<=.12
Meropenem	S	<=1
Minocycline	S	<=4
Piperacillin	S	<=16
Piperacillin/Tazobactam	S	<=16
Tobramycin	S	<=4
Trimethoprim/Sulfamethoxazole	S	<=2/38

**Figure 3:**
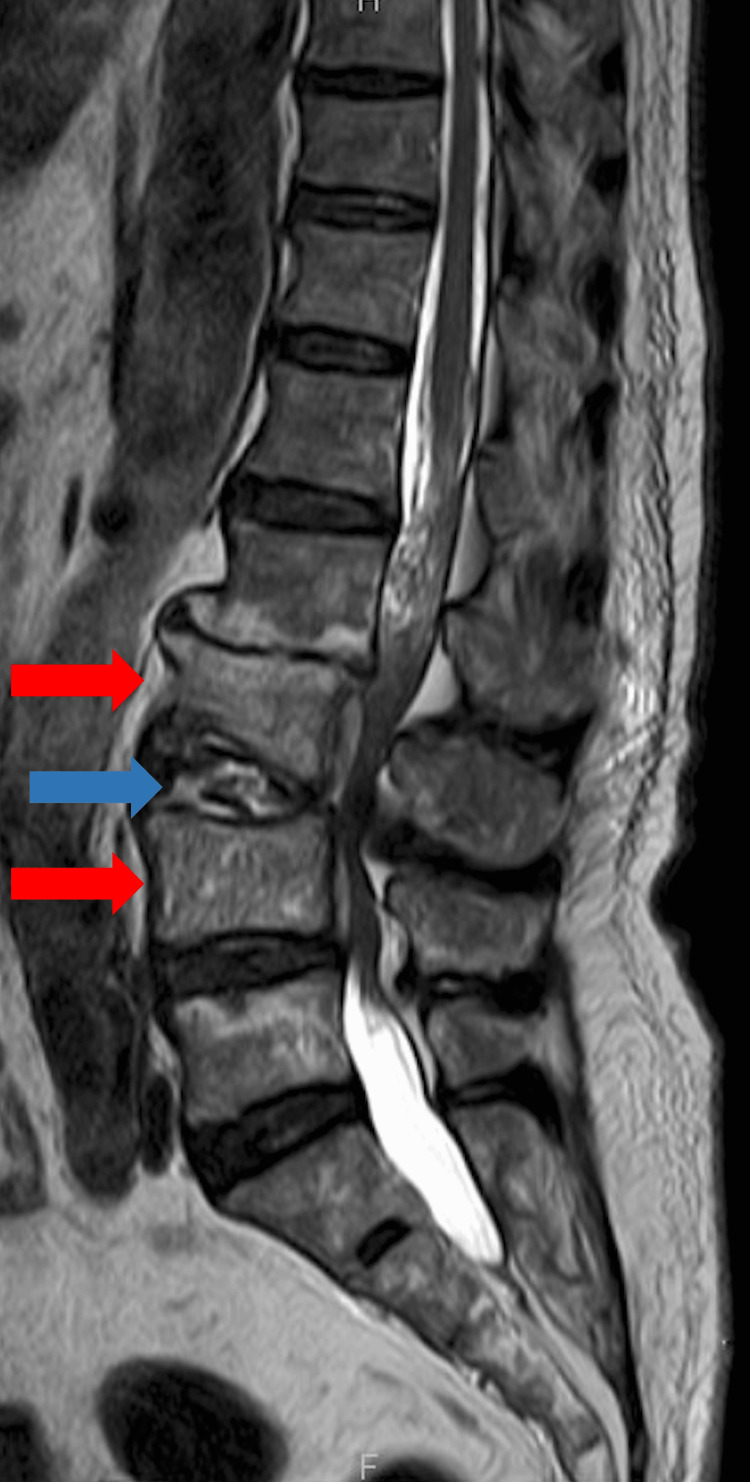
Magnetic resonance imaging of the lumbar spine on day nine showed hyperintensity of the L3 and L4 vertebral bodies (red arrows) and the contiguous disk (blue arrow).

The antibiotics were changed to ceftriaxone and gentamicin, as infectious endocarditis (IE) caused by non-*Haemophilus*, *Aggregatibacter*, *Cardiobacterium*, *Eikenella*, and *Kingella *(HACEK) was considered. However, the patient did not have IE according to the modified Duke criteria, and the blood culture on hospital day eight was negative. His lower back pain persisted, leading to a repeat MRI of the lumbar spine on day nine. It revealed evidence of L3-L4 osteomyelitis with increased signal intensity from the adjacent disks and vertebrae (Figure 3).

Owing to renal failure, gentamicin was discontinued on day 19, and the patient was treated with six weeks of intravenous ceftriaxone, followed by oral levofloxacin. The patient underwent lumbar pedicle screw fixation and laminectomy for residual back pain and was discharged from the hospital on day 90 without any neurological deficits.

## Discussion

This case report concerns a patient with vertebral osteomyelitis caused by *E. coli*, which is not a common pathogen for osteomyelitis like *Staphylococcus aureus* andcoagulase-negative staphylococci. Mohammadreza et al. reviewed 10 cases of *E. coli* spondylodiscitis and found the most common origin of the infection was the urinary tract, accounting for at least 50% of cases [5]. However, our patient had no urological symptoms, and his urine culture was negative. It has been suggested that infectious dissemination to the vertebrae may occur through Batson’s venous plexus, which is a group of valveless paravertebral veins communicating with the pelvic venous plexus [6]. The patient may have been afflicted with vertebral osteomyelitis from spontaneous transient bacteremia after the first fall on his back, with subsequent bacteremia after the second fall, which led to the rupture of the vertebrae and its venous plexus. According to this acute phase, it is believed that his complete blood count on day one was normal since leukocytosis was found the next day. This patient suffered from persistent bacteremia, which is a critical feature of bloodstream infections such as IE or an intravascular source [7]. Our team treated him with ceftriaxone and gentamicin for non-HACEK IE. Nevertheless, his lower back pain persisted, and a repeat MRI led to the diagnosis of vertebral osteomyelitis. It can be useful when the initial test is unable to provide a diagnosis [8]. It is worth noting that Batson’s plexus is a pool of blood; therefore, when a patient suffers from backache and persistent bacteremia is detected, clinicians should keep in mind that vertebral osteomyelitis can be a source of bloodstream infections.

## Conclusions

Here, we present an uncommon case of *E. coli* vertebral osteomyelitis, which presents with persistent bacteremia. The nonspecific symptoms make it challenging to diagnose the disease; however, a repeat MRI could be useful. Vertebral osteomyelitis may be caused even without urinary tract infections through Batson’s venous plexus, which can act as a potential route of infection. The plexus is also known as a pool of blood around vertebrae. Therefore, it is essential for clinicians to suspect vertebral osteomyelitis when bacteremia persists.
